# The evolution of *Lachancea thermotolerans* is driven by geographical determination, anthropisation and flux between different ecosystems

**DOI:** 10.1371/journal.pone.0184652

**Published:** 2017-09-14

**Authors:** Ana Hranilovic, Marina Bely, Isabelle Masneuf-Pomarede, Vladimir Jiranek, Warren Albertin

**Affiliations:** 1 The Australian Research Council Training Centre for Innovative Wine Production, Adelaide, South Australia, Australia; 2 Department of Wine and Food Science, The University of Adelaide, Urbrrae, South Australia, Australia; 3 Unité de recherche Œnologie, Institut de la Science de la Vigne et du Vin, University of Bordeaux, Villenave d'Ornon, France; 4 Bordeaux Sciences Agro, Gradignan, France; 5 ENSCBP, Bordeaux INP, Pessac, France; Institut de Genetique et Microbiologie, FRANCE

## Abstract

The yeast *Lachancea thermotolerans* (formerly *Kluyveromyces thermotolerans*) is a species with remarkable, yet underexplored, biotechnological potential. This ubiquist occupies a range of natural and anthropic habitats covering a wide geographic span. To gain an insight into *L*. *thermotolerans* population diversity and structure, 172 isolates sourced from diverse habitats worldwide were analysed using a set of 14 microsatellite markers. The resultant clustering revealed that the evolution of *L*. *thermotolerans* has been driven by the geography and ecological niche of the isolation sources. Isolates originating from anthropic environments, in particular grapes and wine, were genetically close, thus suggesting domestication events within the species. The observed clustering was further validated by several means including, population structure analysis, F-statistics, Mantel’s test and the analysis of molecular variance (AMOVA). Phenotypic performance of isolates was tested using several growth substrates and physicochemical conditions, providing added support for the clustering. Altogether, this study sheds light on the genotypic and phenotypic diversity of *L*. *thermotolerans*, contributing to a better understanding of the population structure, ecology and evolution of this non-*Saccharomyces* yeast.

## Introduction

The terms ‘yeast’ and ‘*Saccharomyces cerevisiae’* are often used interchangeably. Not surprisingly so; this microorganism, accompanying humans’ progress since Neolithic times [1], is widely used for the production of food, beverages, biofuel and a variety of biochemicals. It is also the best studied eukaryotic model organism, with genome sequences available for hundreds of strains [2–4], and ongoing projects aimed at determining biological functions and genetic interactions of each and every component of its genome [5, 6]. Less is known about other species, commonly referred to as ‘non-conventional’ or ‘non-*Saccharomyces’* yeasts. Scientific interest in them is, however, gaining momentum, as their uncommon physiological, metabolic and cellular functions warrant their further exploration and, ultimately, biotechnological application. One species of remarkable, yet underexplored, biotechnological potential is *Lachancea thermotolerans*.

Formerly known as *Kluyveromyces thermotolerans*, *L*. *thermotolerans* is the type species of the genus *Lachancea* [7]. This genus was proposed by Kurtzman in 2003 to accommodate a group from several different genera showing similarities at the rRNA level. To date, the genus harbours 11 other species: *L*. *cidri*, *L*. *dasiensis*, *L*. *fantastica*, *L*. *fermentati*, *L*. *kluyveri*, *L*. *lanzarotensis*, *L*. *meyersi*, *L*. *mirantina*, *L*. *nothofagi*, *L*. *quebecensis* and *L*. *walti*. From the ecological viewpoint, most *Lachancea* species are ubiquitous [8]. Accordingly, *L*. *thermotolerans* commonly occupies a range of natural and anthropic habitats, including insects, plants, soil and horticultural crops, in particular grapes and wine [9–12]. As so-called protoploid *Saccharomycetaceae*, the *Lachancea* species have diverged from the *S*. *cerevisiae* lineage prior to the ancestral whole genome duplication, and as such offer a complementary model to study evolution and speciation in yeast [13].

Apart from the taxonomic re-classification of *L*. *thermotolerans*, the DNA sequencing era also resulted in extensive genomic information. The nuclear genome of the type strain CBS 6430 is 10.6 Mb and contains 5,350 annotated genes organised in eight chromosomes [13, 14]. Mitochondrial genome sequences are available for 50 strains, and are highly conserved within the species [9]. Despite the ample genomic information, the ploidy of *L*. *thermotolerans* remains controversial; diploid according to some authors [13, 14], haploid according to the others [9, 15].

Another underexplored trait is the peculiar ability of *L*. *thermotolerans* to produce lactic acid during alcoholic fermentation [16]. Lactic acid production is an uncommon metabolic activity among yeasts [17] but it is, however, of great biotechnological interest [18, 19]. The maximum reported lactic acid concentration obtained during *L*. *thermotolerans* alcoholic fermentation is 16.6 g/L [15]. In comparison, wildtype *S*. *cerevisiae* strains in such conditions normally produce only about 0.2–0.4 g/L [18, 19]. While yields obtained by *L*. *thermotolerans* remain insufficient for industrial bulk chemical production, they are of interest for processes in which alcoholic fermentation with concomitant acidification is a benefit; notably winemaking.

Indeed, the use of *L*. *thermotolerans* inocula to partially conduct fermentation is being increasingly explored in winemaking [20–23]. The resultant biological acidification is considered to positively affect the organoleptic quality and microbial stability of the resulting wines [16]. Other positive chemical and sensorial modulations include lower final ethanol content [21], increasingly in demand on the market [24], and improved wine aroma, flavour and mouthfeel [16, 20, 21]. Accordingly, several *L*. *thermotolerans* co-starters are now commercially available to be used in wine fermentations with either simultaneously or sequentially inoculated *S*. *cerevisiae* [16].

Population genetics studies in several yeast species, including *L*. *thermotolerans*, have revealed differentiation of subpopulations according to their geographical and/or ecological origin [25]. In *L*. *thermotolerans*, grouping based on the geographical origin has been determined by the mitochondrial and nucleic DNA analysis of 50 isolates [9]. Nonetheless, information on population diversity, evolution and structure is lacking. In the current study, we explore the relationships of 172 isolates from diverse ecological niches worldwide. Using a 14-loci microsatellite genotyping method, coupled with phenotyping assays, we demonstrate that both geographic localisation and anthropisation have significantly contributed to the diversity and evolution of *L*. *thermotolerans*.

## Materials and methods

### Yeast isolates, culture conditions and DNA preparation

**Fig 1:**
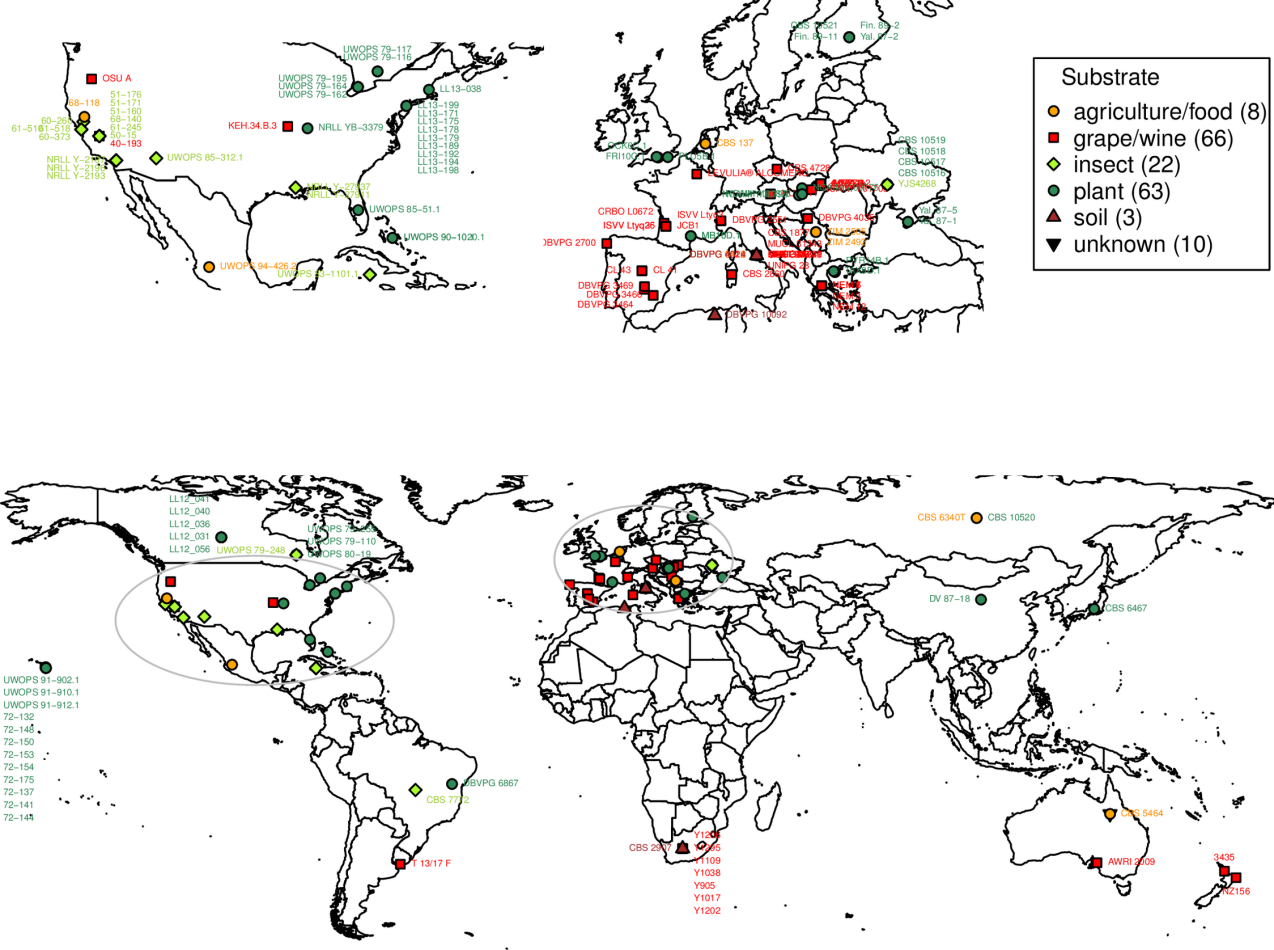
Geographic origin of the genotyped *L*. *thermotolerans* isolates obtained from different substrates. Isolates with unknown origin (see S1 Table) are not represented on the map.

Yeast isolates catalogued as *L*. *thermotolerans* were obtained from multiple yeast culture collections and generous laboratories worldwide. Excluding any obvious issues of selective enrichment inherent to any culture-based study, the sample set represented diverse ecological niches (e.g. oenological environments, plant material, insects) covering a wide geographic span (S1 Table). The isolates were mapped in Fig 1 using R package maps [26]. In addition, the type strains of 11 other *Lachancea* species (S1 Table), were included in the study. Cryogenically stored isolates (-80°C in 25% glycerol) were cultured on YPD plates (1% yeast extract, 2% peptone, 2% glucose and 2% agar) for 3 days at 24°C. DNA template for genotyping was prepared by heating a suspension of approximately 10^7^ cells in 100 μL of 20 mM NaOH at 94°C for 10 minutes, followed by storage at -20°C. For phenotyping purposes, approximately 10^7^ cells were grown for 24 hours at 24°C in 500 μL YPD agitated on a rotary shaker in deep 96-well plate format.

### Microsatellite loci

Microsatellite markers were detected within the genomic sequence of *L*. *thermotolerans* CBS 6340 type strain as described previously [27]. Primers were designed using the ‘Design primers’ tool on the SGD website (http://www.yeastgenome.org/cgi-bin/web-primer). In addition, five microsatellite loci developed by Banilas et al. [15] were included in the study. In order to reduce the cost associated with primer fluorescent labelling, forward primers were tailed on the 5’-end with the M13 sequence as described by Schuelke [28]. Amplification specificity and optimal PCR conditions were assessed for all the loci (Table 1).

**Table 1:** Microsatellite loci for *L*. *thermotolerans* genotyping.

Locus	Chr.	Coordinates	Motif	Primers^b^	Dye	Tm	Number of alleles	Size range	Coding sequence	Function
LT2A	2	610672–610712	ACA	F:TGACAAAAGTTTATCCCCCC	NED	62	24	385–438	XP_002552115	RNA-binding protein
				R:AGCACTGGCGATATCTTGGTT						
LT3A	3	129153–129184	AGC	F:CAGTACCAGCGCCAGTTCTA	PET	60^c^	25	293–352	XP_002552291	peroxin family member
				R:TTCTGTAGCTTGGGGTTGTGT						
LT3B	3	621739–621768	AGC	F:ACAGCAGCAGCAACAGCAA	NED	60^c^	9	86–111	no similarity found	na
				R:TTCGCCAAGCTGCTGATACTA						
LT4A	4	897528–897557	AGA	F:AGAAGGAGGACTCAGCGGATT	NED	60^c^	12	222–260	no similarity found	na
				R:ATGCCTAAGCGAATCAGATGC						
LT5B	5	317191–317225	ATA	F:AACGCTGACGTGCTGAAAGA	FAM	56	10	275–314	no similarity found	na
				R:GAAAGAGGCAGTAACGGATTT						
LT6B	6	134618–134640	ACA	F:TTCCTAGGTCTGGACCTCCAA	PET	60^c^	24	106–161	no similarity found	na
				R:TATTGCTGCTGCTTTTGCTG						
LT7A	7	1417616–1417644	TGT	F:TTTTTTCTTGATGCCCCGGT	FAM	60	10	131–150	XP_002555739	unknown; kinase suppression effect
				R: CGAACTGTGGTTCCTTCACAT						
LT8A	8	638186–638223	TCC	F:TGAAATAGAGTCCCGTGTGAA	PET	62	28	182–240	XP_002556192	vacuolar protein sorting
				R: AAATAACGCAGAAAGCGAGG						
LT8B	8	239222–239256	ATG	F:CAGCATCCGCACAGTAGCTAA	HEX	60^c^	9	261–286	XP_002555998	nuclear DNA helicase
				R:TTATCTCCTTATGCGGGCGTA						
MA2^a^	1	358081–358339	CA	F:AATTTTACGAAGGGAGAGAGGG	NED	60^c^	44	298–358	XP_002551596	bud-site selection nutrient signaling
				R:CTGCTGATGGTTTCTTCTGTGA						
MD3^a^	4	259537–259789	CAA	F:ACAAGAAAGCGAAGGAAAACAG	FAM	62	41	353–485	XP_002552792	unknown; hypothetical ORF
				R: CCCAGTAGAACGTGATTAAGCC						
ME11^a^	5	1381401–1381503	TG	F:CGGTTCTTAGCTTACCAACAGC	HEX	52	30	148–209	XP_002554109	mitotic spindle-associated protein
				R:ACTCGAACAGCCAGAGCTTAAC						
ME4^a^	5	576050–576253	GA	F:TGGCCTCTTCTGTCTTTCCTAA	HEX	60^c^	34	346–421	no similarity found	na
				R:CTCATCAACCAACACACTCCAT						
MH6^a^	8	372940–373089	TGT	F:CTTGCTGTTGTCGTAACCTCTG	PET	62	49	374–566	XP_002556014	ER-associated protein degradation; hypothetical ORF
				R:AATCCCAATAATCTCACACCC						

Chr.—chromosome; Tm—melting temperature.

^a^ Banilas et al. (2016)

^b^ M13 sequence was attached at the 5’ end of the forward primer

c touch-down PCR commenced at Tm + 10°C with a 1°C decrease per cycle (see Materials and Methods).

### Microsatellite amplification

PCRs were carried out in a final volume of 15 μL containing 1 μL of DNA template solution, 1X Taq-&GO (MP Biomedicals, Illkirch, France), 0.05 μM of forward primer, and 0.5 μM of reverse and labelled primer. Universal M13 primers were labelled either with FAM-, HEX-, PET- or NED- fluorescent dyes (Eurofins MWG Operon, Les Ulis, France). Amplifications were performed in an iCycler (Biorad, Hercules, CA, USA) thermal cycler. The program comprised an initial denaturation of 1 minute at 94°C; 30 annealing cycles with 30 seconds at 94°C, 35 seconds at Tm, or Tm +10°C with a 1°C decrease per cycle until Tm was achieved, 30 seconds at 72°C; and a final elongation at 72°C for 10 minutes (Table 1). Upon initial amplification verification by a microchip electrophoresis system (MultiNA, Shimazdu), amplicons were diluted in deionised water (1,200-fold for HEX, 2,400-fold for PET, 3,600-fold for FAM and NED). Amplified fragment sizes varied from 86 to 566 base pairs, allowing for the multiplexing of all the amplicons in formamide. LIZ 600 molecular marker (100-fold dilution) was added to each multiplex, heated for 4 minutes at 94°C. The sizes were of amplicons were then measured on an ABI3730 DNA analyser (Applied Biosystems), and recorded using GeneMarker Demo software v2.4.0 (SoftGenetics).

### Microsatellite data analysis

Microsatellite data, i.e. recorded alleles sizes, were analysed using R software [26]. To examine the genetic relationships between genotyped *L*. *thermotolerans* isolates, a dendrogram was constructed using Bruvo’s distance, particularly well adapted for cases of unknown/multiple ploidy levels [29], and Neighbour Joining (NJ) clustering [30] using poppr [31], ape [32], plotrix [33] and geiger [34] packages. The robustness of the identified clusters was further tested by several means, including node reliability assessment based on the algorithm by Prosperi et al. [35], a dendrogram construction with Bruvo’s distance and UPGMA clustering, and principal component analysis (PCA) of the allelic data using ade4 package [36]. Population differentiation among obtained genetic groups was tested with the fixation index (F_ST_), computed with polysat [37] package. Bootstrapping (n = 100) of the F_ST_ indexes was performed, and confidence intervals were calculated for the obtained values.

Population structure analysis based on the Bayesian approach was performed in R package LEA [38], using non-negative matrix factorization (sNMF) algorithm [39] for estimating individual ancestry coefficients. Models with number of populations (K) ranging from 1 to 40 were tested in 100 repetitions. Two models were selected for graphical representation: (i) K = 12 resulting in the lowest cross-entropy value, and (ii) K = 8 featuring the minimal ancestral population number and statistically equivalent cross-entropy to K = 12 (Kruskal–Wallis (KW) test; alpha = 0.05; package agricolae).

Analysis of molecular variance (AMOVA) was performed to assess whether the genetic distance was significantly explained by the substrate and geographical origin of isolation using the pegas package 0.6 [40] with 1,000 permutations. The relationship between genetic distance and geographic localisation was further verified by Mantel’s test, allowing for the correlation of two distance matrices [41]. A genetic distance matrix obtained from microsatellite data was correlated to a kilometric distance matrix obtained from coordinates of isolation using ade4 and sp packages [42], with the number of permutations set at 1,000.

### Phenotypic analysis

Plate-based assays were performed to assess the growth rate and extent of 132 *L*. *thermotolerans* alongside 11 non-*thermotolerans* strains using different carbon sources and physicochemical conditions. Cell density and viability of pre-established yeast cultures was determined by flow cytometry coupled with propidium iodide DNA staining (Quanta SC MPL, Beckman Coulter, France). Cultures were diluted to 10^5^ viable cells/mL and 2 μL of the obtained dilution was plated onto the appropriate media. All tests were performed in triplicate and, unless otherwise specified, incubated at 24°C. Growth on standard YPD was evaluated at 3 temperatures: 24°C (control), 8°C (lower temperature) and 30°C (higher temperature). In media for testing carbon utilisation, 2% glucose in YPD was substituted with 2% of one of the following carbon sources: fructose, xylose, mannose, galactose and glycerol. Osmotolerance was tested on plates containing 25% and 50% (w/v) of equimolar concentrations of glucose and fructose. Plates were imaged after 3, 6 and, for 8°C condition, 10 days of incubation, and analysed using ImageJ2 software [43]. Upon converting uploaded images into a binary mode (black background, white foreground), colony sizes were determined via pixel density measurements using the ROI (region of interest) function. The colony size from each condition was compared to that on the standard YPD plate incubated at 24°C for 3 or 6 days. Phenotyping data was analysed using R packages gplots, RColorBrewer, plot3D and agricolae [26]. A heatplot and a dendrogram (Euclidean distance and Ward clustering) were constructed to visualise the performance of individual phenotyped isolates. The differences among the determined *L*. *thermotolerans* genetic groups were tested with KW tests and post-hoc multiple comparison of modalities to assess levels of significance (alpha = 0.05).

## Results

### Polymorphic microsatellite markers for *L*. *thermotolerans*

The genomic sequence of *L*. *thermotolerans* type strain CBS 6340 was mined to identify tandem iterations of two or more nucleotides, located on positions other than the 5’-end and 3’-end of the chromosomes to exclude possible (sub)telomeric positions. Primer pairs were designed to amplify microsatellites, and their amplification specificity was ascertained using a sub-panel of 15 *L*. *thermotolerans* isolates using a microchip electrophoresis system MultiNA. Nine loci covering seven of the eight CBS 6340 chromosomes were retained for further analysis, five of these situated within putative coding sequences (Table 1). This set of microsatellites was extended with five markers previously used for *L*. *thermotolerans* genotyping [15]. All 14 markers were tested on 11 non-*thermotolerans* type strains, resulting in a good amplification of several markers (S2 Table). Some of the microsatellites developed for *L*. *thermotolerans* were therefore deemed as potentially suitable for diversity studies of other species belonging to the genus *Lachancea*. Eight loci were amplified in *L*. *quebecensis*, a species very closely related to *L*. *thermotolerans*. Amplification on all loci was, however, exclusive for *L*. *thermotolerans* strains, allowing for taxonomic confirmation at a species level, and thus confirming the identity of the 172 *L*. *thermotolerans* isolates. A comparable number of genotyped isolates originated from anthropic environments and nature: 75 and 88, respectively. Given the importance of the species to oenology, most of the samples from the anthropic milieu were reported as isolated from wine-related environments. Moreover, both anthropic and natural sub-groups comprised representatives from each continent/region of isolation.

All markers were polymorphic, with the number of alleles varying between 9 for loci LT3B and LT8B, and 49 for locus MH6 (Table 1). Interestingly, a single allele per locus was obtained for all tested isolates. Of the 172 isolates, 136 distinct genotypes were observed, confirming the discriminatory power of the microsatellite analysis.

### Genetic proximity and divergence between *L*. *thermotolerans* isolates

**Fig 2:**
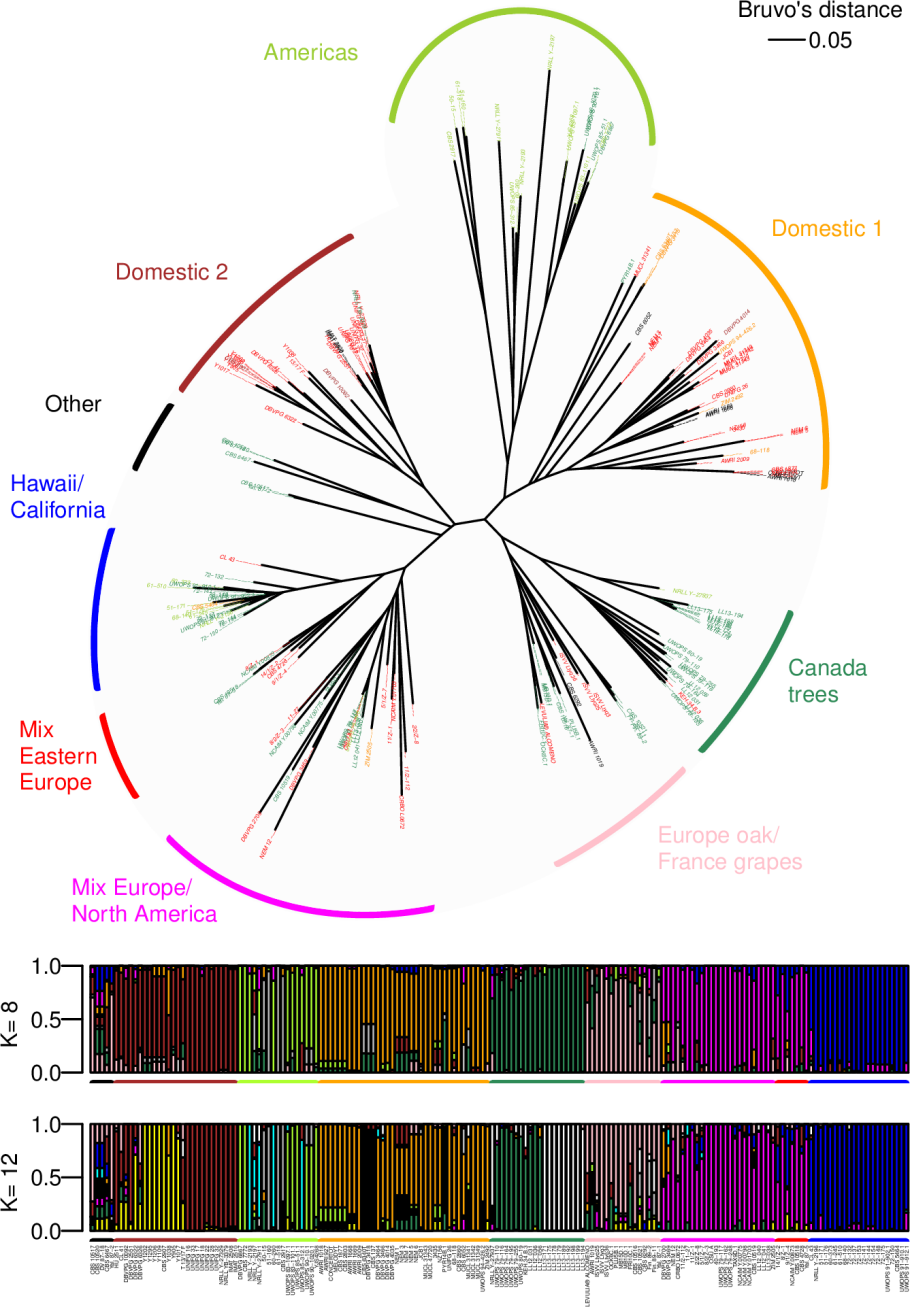
Genetic relationships between 172 *L*. *thermotolerans* isolates determined using 14 microsatellite makers. Colour-coding of isolates corresponds to isolation substrate, as per Fig 1. (A) Dendrogram constructed using Bruvo’s distance and NJ clustering. (B) Barplot representing population structure (K = 8 and K = 12). The posterior probability (y-axis) of assignment of each isolate (vertical bar) to inferred ancestral populations is shown with different colours.

Genetic relationships between *L*. *thermotolerans* isolates were further examined using Bruvo’s distance and the NJ clustering method. The resulting dendrogram (Fig 2) enabled the visualisation and delineation of genetic groups. Some groups mainly comprised isolates originating from natural environments, grouped together based on their origin. One such group, ‘Americas’, consisted of 17 isolates mainly from natural habitats in the Americas (15/17), i.e. southern USA (9/17), Caribbean (4/17) and Brazil (2/17). A second ‘wild’ group, ‘Canada trees’, contained 20 North American isolates of which 18 were found to originate from plant material (*Quercus sp*. and *Prunus sp*.) across Canada. The third wild group (‘Hawaii/California’) harboured 21 isolates from Hawaii (12/21) and California (7/21), sourced from cacti and insects, respectively. Interestingly, identical genotypes could be observed among Hawaiian samples collected from the same habitat with a two-decade temporal span. Isolates 72_148 and 72_175 were collected approximately 20 years prior to the UWOPS 91–902.1, thus indicating the persistence of certain clonal variants. Finally, two separate, albeit small, clusters with tree exudate isolates from Eurasia were differentiated (‘Other’). In addition to ‘wild’ groups, genetic proximity of isolates originating predominantly from anthropic habitats could also be observed. These ‘domesticated’ isolates were, in fact, grouped in two separate clusters. The larger group (‘Domestic 1’) consisted of 36 isolates, the majority from grapes and wine. The 23 oenological samples showed diverse geographic origin; two isolates from New Zealand (NZ156, 3435) and one from Australia (AWRI 2009) clustered closely to 20 European isolates, mostly from the Mediterranean region. It also included six isolates from agriculture and food-related environments from more distant geographical origins, i.e. Russia (CBS 6340T), Europe (CBS 137, DBVPG 3418, ZIM 2492) and North America (68_118, UWOPS 94–426.2). The second ‘domesticated’ group, ‘Domestic 2’ contained 21 grape/wine representatives from different continents, including Europe (Italy, Spain, Austria), Africa (South Africa) and Americas (USA, Uruguay). The remaining two South African isolates from soil (CBS 2907, DBVPG 10092) also clustered in this group, as well as the two isolates of unknown origin (IMAT 2508, IMAT 2510). The remaining genetic clusters were mixed with regards to the location and/or substrate of isolation of their constituents. Seven isolates from ‘Mix Eastern Europe’ formed one such branch. Four of these were isolated from grapes, and three from other plant material (*Quercus sp*. and *Betula sp*.). These clustered close to a group with a total of 24 isolates from Europe (16/24) and North America (8/24), with the representatives of oenological (13/24) and natural habitats (9/24) from both continents, i.e. ‘Mix Europe/North America’. In addition to 12 European oak isolates, the last mixed group (‘Europe oak/France grapes’) encompassed four isolates associated with grapes originating from two French wine regions (i.e. Burgundy and Bordeaux), an Australian and an isolate of unknown origin.

### Validation of observed clustering

**Fig 3:**
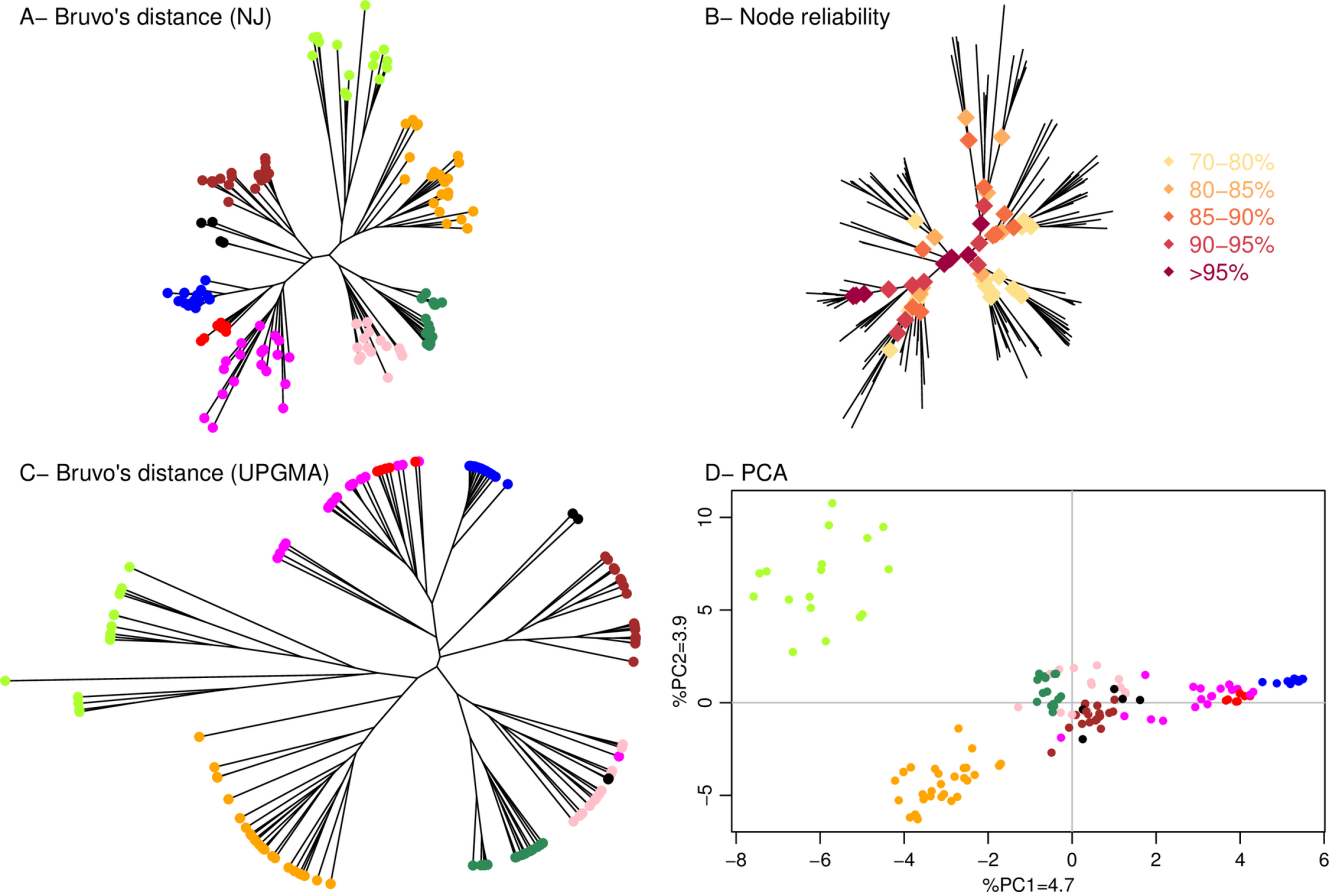
Genetic clustering of 172 *L*. *thermotolerans* isolates determined using 14 microsatellite makers. Each dot represents a genotype, with colours corresponding to determined genetic groups as per Fig 2. (A) Dendrogram constructed Bruvo’s distance and NJ clustering. (B) Reliability assessment of the nodes of the dendrogram constructed using Bruvo’s distance and NJ clustering. (C) Dendrogram constructed Bruvo’s distance and UPGMA clustering. (D) PCA of the allelic data.

Several approaches were used to validate the proposed clustering identified on the Bruvo’s NJ dendrogram (Fig 3). As classical bootstrapping is poorly reliable with microsatellite data, the Prosperi et al. [35] algorithm-based reliability assessment was used to test the robustness of the tree nodes. The reliability values of all major tree nodes exceeded 70% (i.e. bootstrap support > 70; Fig 3B), thus strongly supporting the observed clustering. Next, an UPGMA algorithm was used as an alternative to NJ clustering to plot Bruvo’s distance matrix. Both clustering methods resulted in largely consistent genetic grouping (Fig 3C), albeit ‘Mix Eastern Europe’ clustered among the ‘Mix Europe/North America’ group on the UPGMA dendrogram. A congruent separation of genetic groups could also be observed on the PCA plot of the genetic polymorphism data (Fig 3D), showing a co-localisation of the ‘Mix Eastern Europe’ and ‘Mix Europe/North America’ group, and a suitably resolved partitioning of other groups.

In order to evaluate the differentiation of these populations, a pairwise fixation index F_ST_ was calculated for eight genetic groups (Table 2), as two minor groups (‘Other’) were excluded due to insufficient population size. Overall, a significant differentiation between populations was suggested, with the lowest pairwise F_ST_ value between the ‘Mix Eastern Europe’ and ‘Mix Europe/North America’ clusters, in accord with previous observations. Conversely, ‘Hawaii/California’ was the most differentiated population, followed by the ‘Canada trees’. Interestingly, a comparably low degree of differentiation was obtained between ‘Domestic 2’ and ‘Mix Eastern Europe’ and ‘Americas’ groups, while ‘Domestic 1’ had the lowest pairwise F_ST_ with ‘Europe oak/France grapes’ group.

**Table 2:** Pairwise F_ST_ distance matrix. F_ST_ values are given in the upper matrix, whereas the lower matrix indicates bootstrap values and, in brackets, associated confidence intervals.

	Hawaii /California	Domestic 2	Canada trees	Americas	Mix Europe/North America	Domestic 1	Europe oak/France grapes	Mix Eastern Europe
**Hawaii /California**	na	0.404	0.495	0.413	0.322	0.466	0.425	0.348
**Domestic 2**	0.404 (0.280–0.440)	na	0.28	0.205	0.271	0.28	0.227	0.204
**Canada trees**	0.495 (0.318–0.495)	0.28 (0.084–0.280)	na	0.26	0.31	0.342	0.218	0.319
**Americas**	0.413 (0.413–0.579)	0.205 (0.205–0.400)	0.260 (0.212–0.478)	na	0.272	0.273	0.216	0.258
**Mix Europe/North America**	0.322 (0.322–0.522)	0.271 (0.248–0.371)	0.310 (0.204–0.420)	0.272 (0.241–0.349)	na	0.291	0.238	0.116
**Domestic 1**	0.466 (0.429–0.531)	0.280 (0.261–0.37)	0.342 (0.177–0.392)	0.273 (0.273–0.420)	0.291 (0.256–0.347)	na	0.225	0.256
**Europe oak/France grapes**	0.425 (0.339–0.482)	0.227 (0.227–0.354)	0.218 (0.127–0.330)	0.216 (0.216–0.346)	0.238 (0.172–0.269)	0.225 (0.225–0.331)	na	0.263
**Mix Eastern Europe**	0.348 (0.348–0.500)	0.204 (0.204–0.326)	0.3188 (0.185–0.407)	0.258 (0.205–0.315)	0.116 (0.116–0.314)	0.256 (0.203–0.288)	0.263 (0.189–0.294)	na

Population structure analysis was further conducted to infer ancestral populations (Fig 2B). The number of populations (K) ranged from 1 to 40. The absolute lowest cross-entropy values were found for K = 12, but the cross-entropy values were statistically equivalent (KW test) for K = 8 and up to K = 20 (S1 Fig). Among the ‘wild’ groups, the ‘Hawaii/California’ isolates were assigned to a distinct single ancestry, regardless of the total number of populations. The group of ‘Americas’ isolates, conversely, showed less homogeneity with multiple ancestries. A single and a dual ancestry was indicated for the ‘Canada trees’ group under the K = 8 and K = 12 scenario, respectively. This also seemed to be the case for the ‘Domestic’ groups of isolates. The two closely related mixed groups (‘Mix Eastern Europe’, ‘Mix Europe/North America’) showed similar population structure and a common ancestry, separate to that of ‘Europe oak/France grapes’ group. All these groups had a proportion of mixed origin isolates, especially in K = 12 simulation model. Overall, such results were in strong accord with the previous analysis (dendrograms, PCA, etc.).

To determine whether, and to what extent, the isolation substrate and geographic origin have significantly shaped *L*. *thermotolerans* genetic variation, an AMOVA was performed. The genetic distance was tested in relation to the continent/region of provenance (S1 Table), and habitat types grouped either as ‘domestic’ or ‘wild’. Both geographic location and habitat were found to be significant, explaining 20.85% and 13.58% of variation, respectively (P < 0.0001). The relationship between genetic distance and geography was further confirmed by Mantel’s test, indicating a significant link between the genetic and kilometric distance matrices of the whole sample set (P = 0.00009), samples from Europe (P = 0.00009) and Americas (P = 0.00019).

### Phenotypic variability of the tested sample set

**Fig 4:**
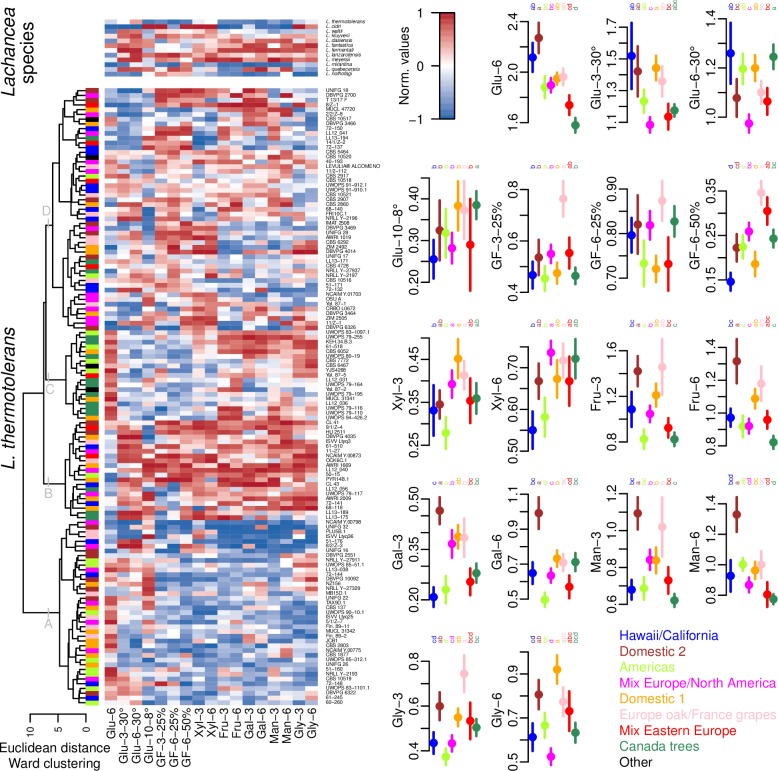
Phenotypic performance tested on plates using different carbon sources and physicochemical conditions. **Dendrogram constructed with Euclidean distance and Ward clustering using normalised values of obtained growth of 132 *L*. *thermotolerans* and 11 non-*thermotolerans* strains in tested conditions, and/or a corresponding heatplot (left). Comparison of phenotypic performance at a genetic group level (right).** Glu–glucose, GF–equimolar mixture of glucose and fructose, Xyl–xylose, Fru–fructose, Gal–galactose, Man–mannose, Gly–glycerol; unless otherwise specified, carbon sources were supplemented in concentration of 2%, and incubation temperature was 24°C; numbers 3, 6 and 10 refer to the incubation duration. No quantifiable growth was observed for ‘GF-3-50%’, ‘G-3-8°’ and ‘G-6-8°’ modalities, thus not included graphical representation. Colours of the represented individuals/genetic groups correspond to Figs 2 and 3. Dots and bars represent normalised growth means and ranges, respectively, and letters denote significance levels between genetic groups (KW tests; alpha = 0.05).

Phenotyping assays testing growth performance of 132 *L*. *thermotolerans* and 11 non-*thermotolerans* strains showed substantial variability at the species/strain level (Fig 4). Using the phenotypic dataset, a dendrogram was built using Euclidean distance and Ward’s clustering. In general, one cluster of isolates (A) displayed a lower degree of growth on all substrates and conditions except glucose, with a subset of isolates growing well at 8°C. Conversely, the second group (B) showed better growth on all tested substrates. Group C was less prolific at lower and higher temperatures, under osmotic stress and on xylose, compared to fructose, galactose, mannose and glycerol. The largest and the most variable group, D, contained isolates generally exhibiting osmotolerance. It featured a subset with lesser growth at 30°C and on glycerol, and another with an extensive growth on xylose.

Several findings regarding the comparison of phenotypic performance at the genetic group level warrant highlighting. Firstly, the two ‘domestic’ groups (‘Domestic 1’ and ‘Domestic 2’) were among groups displaying superior growth aptitude in the majority of tested conditions. Next, the ‘Europe oak/France grapes’ group, followed by the ‘Mix Eastern Europe’ group, grew best on plates testing osmotolerance. Interestingly, among natural isolates, these groups contained representatives sourced from grape musts in Sauternes and mummified grapes in Tokay, i.e. high sugar concentration substrates. Finally, a superior growth of ‘Canada trees’ isolates was observed at 8°C compared to all other groups, without being impaired at 30°C.

## Discussion

Despite the rapid progress in DNA sequence analysis, microsatellites, rather than being obsolete, represent an informative, cost-effective tool for genotyping purposes, well adapted to large sample sizes. In fact, few genetic markers, if any, have found such widespread application for population diversity, ecology and evolution studies [44]. In yeasts, they were successfully applied to elucidate population structure of several species, including *S*. *cerevisiae* [45, 46], *S*. *uvarum* [47], *Torulaspora delbrueckii* [27], *Starmerella bacillaris* [48], *Hanseniaspora uvarum* [49] and *Brettanomyces bruxellensis* [50]. A set of five microsatellites has thus far been developed for *L*. *thermotolerans* [15], hereby extended with nine novel loci. This improved multilocus genotyping method was used on 172 isolates of diverse geographic and ecological origin, shedding light on *L*. *thermotolerans* diversity and population structure.

The resultant clustering revealed that the evolution of *L*. *thermotolerans* has been driven by the geography and the ecological niche of the isolation sources. This observation was subsequently confirmed with F-statistic, Mantel’s test and AMOVA results. A link between phylogeny and geography has previously been reported for this species; a differentiation in relation to habitat has, conversely, not been established [9]. While the overall clustering remains congruent between both studies, the enlarged sample size with a balanced number of natural and anthropic isolates might account for such disparity. Indeed, the current study provides a compelling case for domestication occurrence within *L*. *thermotolerans* population, implying selection, intended or not, of variants related to anthropic environments. Scientific interest in microbial domestication is on the rise, and has been confirmed for *S*. *cerevisiae* [46, 51] and, more recently, for *T*. *delbrueckii* [27]. In each of these species, a separate wine-related lineage was detected, along with groups of individuals associated with other bioprocesses (e.g. baking, dairy, bioethanol etc.). Strikingly, two separate structured (F_ST_ = 0.280) *L*. *thermotolerans* domestic sub-populations with distant ancestries were hereby resolved, indicating multiple domestication events. Both clades were comprised largely of wine-related samples, with isolates from other anthropic environments (i.e. milk, distilling, fruits) clustering among the oenological ones. This suggests that, while some strains occupy diverse anthropic niches, further differentiation has not been achieved, although a larger sample subset (i.e. more isolates from anthropic environments other than grapes and wine) is required to confirm this hypothesis. Persistence in the grape and wine-related ecosystems involves survival in rather extreme conditions, ranging from the frequent exposure to agrochemicals, especially sulphur and copper, in vineyards, to the particularly harsh conditions during winemaking. Accumulated sugars exert the initial hyperosmotic stress, while fermentation leads to the accumulation of ethanol concentrations toxic for the yeast cells [52]. Several other (a)biotic stressors are also imposed, including oxygen and nutrient depletion, unfavourable physicochemical conditions (low pH, temperature shocks, SO_2_ addition, etc.) and inhibitory microbial interactions [16, 52]. It is therefore plausible that such selective environments have led to differentiation of the two domestic clusters. Interestingly, both domestic clusters encompassed representatives from Europe and so-called ‘New World’ winegrowing countries (Australia and New Zealand for ‘Domestic 1’; Americas and South Africa for ‘Domestic 2’), hinting at a contributing role of viti-vinicultural expansion towards a wider dispersal of some genotypes. This is in line with well-established expansion of grape-growing and winemaking practices from the Mediterranean basin to, ultimately, all wine regions across the globe [53].

Groups harbouring isolates from both cultivated and natural ecosystems, on the other hand, suggest the inter-connectivity of different ecological niches. A free flow of individuals can lead to absence of differentiation between cultivated and wild environments within a limited geographic span, as previously reported for *S*. *cerevisiae* communities in New Zealand [54] and USA [55]. The isolation proximity of certain samples within ‘mixed’ groups supports this observation, in particular within the ‘Mix Eastern Europe’ cluster, and among ‘Mix Eastern Europe’ and some ‘Mix Europe/North America’ genotypes. Common vectors for the inferred yeast dissemination between different ecological reservoirs are insects like bees, wasps and fruit flies [56, 57], while dispersal over a larger geographical span, also seen among mixed groups, requires other carriers—likely birds [58] and humans. The carryover between ecosystems is also indicated within *L*. *thermotolerans* ‘natural’ groups, in particular within the ‘Hawaii/California’ group. Given the spatial isolation of the Hawaiian islands, and their volcanic origin, migration events are to be presumed. This may also be the case with the seemingly most heterogeneous cluster of American isolates. Altogether, this dataset paints a comprehensive picture of *L*. *thermotolerans* evolution being shaped by anthropisation and geographic origin, as well as the macroorganism-mediated flux between different ecosystems.

Colonisation of a given niche is known to lead to evolutionary differentiation, harnessing adaptation to specific environmental conditions [25]. A set of plate-based growth assays was therefore carried out to examine whether the genotypic diversity is echoed on a phenotypic level. Interestingly, the overall prolific growth of ‘domestic’ groups could be observed, that might have contributed to their inter-continental dispersal and persistence in a large range of anthropic-related environments. Evidence for a narrower ecological adaptation was also suggested; e.g. a superior growth of Canadian isolates at 8°C, possibly reflecting their adaptation to (sub)boreal climate conditions. Overall, a marked intra-specific diversity at a phenotypic level could be observed, to a degree supporting genetic differentiation. Further experimental verification of genotype-phenotype inter-groups relationships, however, is required to support such claims.

Apart from population structure, microsatellites can be used to elucidate life cycle of studied organisms [27, 59]. The ploidy of *L*. *thermotolerans* is controversial. Due to its sporulation ability, it was originally deemed to be a diploid species [14]. Conversely, Freel et al. [9] have reported most natural isolates to be haploid, in line with the single-allele microsatellite patterns observed in Banilas et al. [15]. As only one allele per locus was recorded on all 14 microsatellite loci for all 172 isolates used in this study, additional support for the haploid status of *L*. *thermotolerans* is provided. Nonetheless, absence of heterozygosity and/or diploidisation of haploids cannot be excluded. Further elucidation of the species’ life cycle particularities is thus still required, as well as establishing sporulation conditions, mating patterns, occurrence and distribution of heterothallic and/or homothallic variants, and their potential implications for the diversity and evolution of the species.

In conclusion, this study provides a valuable insight into the genotypic and phenotypic diversity of *L*. *thermotolerans*, contributing to a better understanding of population structure, ecology and the evolution of this remarkable yeast species.

## Supporting information

S1 Table List of microorganisms used in the current study. Genotyping was undertaken on all the listed *L*. *thermotolerans* isolates, and phenotyping on isolates/strains in bold. Italicised isolates were obtained in the isolated DNA format.
(PDF)

S2 Table Amplification of *L*. *thermotolerans* microsatellite markers on *Lachancea* species. Numbers are coded as following: 0—no amplification, 1—faint band, 2—medium intensity band, 3—full intensity band as determined using a microchip electrophoresis system.
(PDF)

S1 Fig Kruskal–Wallis test of cross-entropy values for numbers simulated ancestral populations. (PDF)
