# Supplementary material for: The evolution of Lachancea thermotolerans is driven by geographical determination, anthropisation and flux between different ecosystems
Source: PLoS One. 2017 Sep 14;12(9):e0184652. doi: 10.1371/journal.pone.0184652 (PMC5599012; doi:10.1371/journal.pone.0184652)
Supplement: S1 Table — Genotyping was undertaken on all the listed L. thermotolerans isolates, and phenotyping on isolates/strains in bold. Italicised isolates were obtained in the isolated DNA format. (PDF) [file pone.0184652.s001.pdf]

Species	Strain	Source	Isolation origin	Continent/region	Isolation habitat	Habitat type	Genetic group	Reference
L. thermotolerans	3435	Auckland University	New Zealand	Australia/Oceania	grapes	grape/wine	Domestic 1	na
L. thermotolerans	**11/2-112**	University of Debrecen	Slovakia, Mala Trna	Europe	grapes, mummified	grape/wine	Mix Europe/N. America	Sipiczki 2016 [a]
L. thermotolerans	**11/Z-1**	University of Debrecen	Slovakia, Mala Trna	Europe	grapes, mummified	grape/wine	Mix Europe/N. America	Sipiczki 2016 [a]
L. thermotolerans	11-27	University of Debrecen	Slovakia, Mala Trna	Europe	grapes, mummified	grape/wine	Mix Europe/N. America	Sipiczki 2016 [a]
L. thermotolerans	**14/1/Z-2**	University of Debrecen	Hungary, Erdöbénye	Europe	grapes, mummified	grape/wine	Mix E. Europe	Sipiczki 2016 [a]
L. thermotolerans	**2/2/Z-8**	University of Debrecen	Hungary, Sárazsadány	Europe	grapes, mummified	grape/wine	Mix Europe/N. America	Sipiczki 2016 [a]
L. thermotolerans	**5/1/Z-7**	University of Debrecen	Hungary, Vinicky	Europe	grapes, mummified	grape/wine	Mix Europe/N. America	Sipiczki 2016 [a]
L. thermotolerans	**8/2/Z-3**	University of Debrecen	Slovakia, Černochov	Europe	grapes, mummified	grape/wine	Mix Europe/N. America	Sipiczki 2016 [a]
L. thermotolerans	**8/Z-1**	University of Debrecen	Slovakia, Černochov	Europe	grapes, mummified	grape/wine	Mix E. Europe	Sipiczki 2016 [a]
L. thermotolerans	9/1/Z-4	University of Debrecen	Slovakia, Mala Trna	Europe	grapes, mummified	grape/wine	Mix E. Europe	Sipiczki 2016 [a]
L. thermotolerans	**40-193**	Phaff YCC	USA, California	North America	grapes, cv. Alicante Bouchet	grape/wine	Mix Europe/N. America	na
L. thermotolerans	**50-15**	Phaff YCC	USA, Southern California, Pinon Flats, San Jacinto Mnts.	North America	*Drosophila pseudoobscura*	insect	Americas	na
L. thermotolerans	**51-160**	Phaff YCC	USA, California, Aspen Valley, Yosemite area	North America	*Drosophila azteca*	insect	Americas	na
L. thermotolerans	**51-171**	Phaff YCC	USA, California, Mather, Yosemite area	North America	*Drosophila pseudoobscura*	insect	Hawaii/California	na
L. thermotolerans	**51-176**	Phaff YCC	USA, California, Mather, Yosemite area	North America	*Aulacigaster sp.*	insect	Hawaii/California	na
L. thermotolerans	**60-260**	Phaff YCC	USA, UCD campus	North America	*Aulacigaster sp.*on *Ulmus carpinifolia* exudate	insect	Americas	na
L. thermotolerans	60-373	Phaff YCC	USA, Lake Beryessa area	North America	*Drosophila pseudoobscura*	insect	Hawaii/California	na
L. thermotolerans	**61-245**	Phaff YCC	USA, North California Pacific Coast, Gualala River area	North America	*Aulacigaster sp.*	insect	Americas	na
L. thermotolerans	**61-510**	Phaff YCC	USA, Lake Beryessa area	North America	*Drosophila pseudoobscura*	insect	Hawaii/California	na
L. thermotolerans	**61-518**	Phaff YCC	USA, Lake Beryessa area	North America	*Drosophila melanogaster*	insect	Americas	na
L. thermotolerans	**68-118**	Phaff YCC	USA, Yuba City	North America	conveyer belt scrapings (Sunsweet Prune)	agriculture/food	Domestic 1	na
L. thermotolerans	**68-140**	Phaff YCC	USA, California	Australia/Oceania	bees	insect	Hawaii/California	na
L. thermotolerans	**72-132**	Phaff YCC	Hawaii, Ahumoa, Saddle Rd area	Australia/Oceania	*Myoporum sandwicens*exudate	plant	Hawaii/California	na

L. thermotolerans	**72-137**	Phaff YCC	Hawaii, Ahumoa, Saddle Rd area	Australia/Oceania	*Myoporum sandwicens*exudate	plant	Hawaii/California	na
L. thermotolerans	**72-141**	Phaff YCC	Hawaii, Ahumoa, Saddle Rd area	Australia/Oceania	*Myoporum sandwicens*exudate	plant	Hawaii/California	na
L. thermotolerans	**72-144**	Phaff YCC	Hawaii, Ahumoa, Saddle Rd area	Australia/Oceania	*Myoporum sandwicens*exudate	plant	Hawaii/California	na
L. thermotolerans	**72-148**	Phaff YCC	Hawaii, Ahumoa, Saddle Rd area	Australia/Oceania	*Myoporum sandwicens*exudate	plant	Hawaii/California	na
L. thermotolerans	**72-150**	Phaff YCC	Hawaii, Ahumoa, Saddle Rd area	Australia/Oceania	*Myoporum flux* clear drip	plant	Hawaii/California	na
L. thermotolerans	72-153	Phaff YCC	Hawaii, Ahumoa, Saddle Rd area	Australia/Oceania	*Myoporum flux* tacky gum	plant	Hawaii/California	na
L. thermotolerans	72-154	Phaff YCC	Hawaii, Ahumoa, Saddle Rd area	Australia/Oceania	*Myoporum sandwicens*exudate	plant	Hawaii/California	na
L. thermotolerans	72-175	Phaff YCC	Hawaii, Ahumoa, Saddle Rd area	Australia/Oceania	*Myoporum sandwicens*exudate	plant	Hawaii/California	na
L. thermotolerans	AWRI 1018	AWMCC	na	na	na	na	Domestic 1	na
L. thermotolerans	**AWRI 1019**	AWMCC	na	na	na	na	Europe oak/France grapes	na
L. thermotolerans	AWRI 1668	AWMCC	na	na	na	na	Domestic 1	na
L. thermotolerans	**AWRI 1669**	AWMCC	na	na	na	na	Domestic 1	na
L. thermotolerans	**AWRI 2009**	AWMCC	Australia, South Australia	Australia/Oceania	grapes	grape/wine	Domestic 1	na
L. thermotolerans	AWRI 927	AWMCC	Italy	Europe	grapes	grape/wine	Domestic 1	na
L. thermotolerans	**CBS 10516**	CBS-KNAW	Ukraine	Europe	*Quercus sp.* exudate	plant	Europe oak/France grapes	na
L. thermotolerans	**CBS 10517**	CBS-KNAW	Ukraine	Europe	*Quercus sp.* exudate	plant	Other	na
L. thermotolerans	**CBS 10518**	CBS-KNAW	Ukraine	Europe	*Quercus sp.* exudate	plant	Mix E. Europe	na
L. thermotolerans	**CBS 10519**	CBS-KNAW	Ukraine	Europe	*Quercus sp.* exudate	plant	Mix Europe/N. America	Freel et al. 2014 [b]
L. thermotolerans	**CBS 10520**	CBS-KNAW	Russia	Russia/Asia	*Quercus sp.* exudate	plant	Other	Freel et al. 2014 [b]
L. thermotolerans	**CBS 10521**	CBS-KNAW	Finland	Europe	*Quercus sp.* exudate	plant	Europe oak/France grapes	Freel et al. 2014 [b]
L. thermotolerans	**CBS 137**	CBS-KNAW	Netherlands	Europe	date	agriculture/food	Domestic 1	Freel et al. 2014 [b]
L. thermotolerans	**CBS 1877**	CBS-KNAW	Italy	Europe	grapes	grape/wine	Domestic 1	na
L. thermotolerans	**CBS 2803**	CBS-KNAW	Italy	Europe	grapes	grape/wine	Domestic 1	na
L. thermotolerans	**CBS 2860**	CBS-KNAW	Italy, Sardinia	Europe	grape must	grape/wine	Domestic 1	Freel et al. 2014 [b]
L. thermotolerans	**CBS 2907**	CBS-KNAW	South Africa	Africa	soil	soil	Domestic 2	Freel et al. 2014 [b]
L. thermotolerans	**CBS 2917**	CBS-KNAW	na	na	*Drosophila sp.*	insect	Hawaii/California	na
L. thermotolerans	**CBS 4728**	CBS-KNAW	former Czechoslovakia	Europe	grapes	grape/wine	Mix E. Europe	Freel et al. 2014 [b]
L. thermotolerans	**CBS 5464**	CBS-KNAW	Australia	Australia/Oceania	cotton seed	agriculture/food	Hawaii/California	na
L. thermotolerans	**CBS 6052**	CBS-KNAW	na	na	na	na	Domestic 1	na
L. thermotolerans	**CBS 6292**	CBS-KNAW	Australia	Australia/Oceania	na	na	Europe oak/France grapes	na

L. thermotolerans	**CBS 6340T**	CBS-KNAW	Russia	Russia/Asia	mirabelle plum conserve	agriculture/food	Domestic 1	Freel et al. 2014 [b], Naumova et al. 2007 [c]
L. thermotolerans	**CBS 6467**	CBS-KNAW	Japan	Russia/Asia	tree exudate	plant	Other	na
L. thermotolerans	**CBS 7772**	CBS-KNAW	Brazil	South/Central America	*Uca sp.*	insect	Americas	Freel et al. 2014 [b]
L. thermotolerans	**CL 41**	University of Leon	Spain	Europe	grapes	grape/wine	Domestic 2	na
L. thermotolerans	**CL 43**	University of Leon	Spain	Europe	grapes	grape/wine	Hawaii/California	na
L. thermotolerans	CONCERTO™	CHR Hansen	'Mediterranean country''	na	na	na	Domestic 1	na
L. thermotolerans	**CRBO L0672**	CRBOeno	France	Europe	grapes, fermentation	grape/wine	Mix Europe/N. America	na
L. thermotolerans	**DBVPG 10092**	DBVPG	Algeria	Africa	soil, apple orchard	soil	Domestic 2	na
L. thermotolerans	**DBVPG 2551**	DBVPG	Italy, Piemonte	Europe	wine cv. Barbera	grape/wine	Domestic 2	Freel et al. 2014 [b]
L. thermotolerans	**DBVPG 2700**	DBVPG	Spain, La Mancha, Campo de Santiago	Europe	grapes cv. Airen	grape/wine	Mix Europe/N. America	Freel et al. 2014 [b]
L. thermotolerans	DBVPG 3418	DBVPG	Italy	Europe	milk	agriculture/food	Domestic 1	Freel et al. 2014 [b]
L. thermotolerans	DBVPG 3464	DBVPG	Spain, La Mancha, Valdepenas	Europe	grapes	grape/wine	Domestic 1	Freel et al. 2014 [b]
L. thermotolerans	**DBVPG 3466**	DBVPG	Spain, La Mancha, La Encomienda	Europe	grapes	grape/wine	Domestic 1	Freel et al. 2014 [b]
L. thermotolerans	**DBVPG 3469**	DBVPG	Spain, La Mancha, Manzanares	Europe	grapes	grape/wine	Mix Europe/N. America	Freel et al. 2014 [b]
L. thermotolerans	**DBVPG 4014**	DBVPG	Italy	Europe	caverns	soil	Domestic 1	Freel et al. 2014 [b]
L. thermotolerans	**DBVPG 4035**	DBVPG	ex Yugoslavia	Europe	grapes, must	grape/wine	Domestic 1	Freel et al. 2014 [b]
L. thermotolerans	**DBVPG 6322**	DBVPG	Italy	Europe	grapes	grape/wine	Domestic 2	na
L. thermotolerans	**DBVPG 6326**	DBVPG	Italy	Europe	grapes, raisins	grape/wine	Domestic 2	na
L. thermotolerans	DBVPG 6867	DBVPG	Brazil	South/Central America	*Pilosocereus arrabida*	plant	Americas	Freel et al. 2014 [b]
L. thermotolerans	DV 87-18	na	"Far East"	Russia/Asia	*Quercus sp.*exudate	plant	Other	Naumova et al. 2007 [c]
L. thermotolerans	**Fin. 89-11**	na	Finland	Europe	*Quercus sp.*exudate	plant	Europe oak/France grapes	Naumova et al. 2007 [c]
L. thermotolerans	**Fin. 89-2**	na	Finland	Europe	*Quercus sp.*exudate	plant	Europe oak/France grapes	Freel et al. 2014 [b], Naumova et al. 2007 [c]
L. thermotolerans	**FRI10C.1**	NCYC	UK, Fritham, New Forest	Europe	*Quercus sp.*	plant	Europe oak/France grapes	Robinson et al. 2016 [d]
L. thermotolerans	**HU 2511**	BOKU	Austria	Europe	grapes	grape/wine	Domestic 2	na
L. thermotolerans	**IMAT 2508**	IMAT	na	na	na	na	Domestic 2	na
L. thermotolerans	IMAT 2510	IMAT	na	na	na	na	Domestic 2	na
L. thermotolerans	**ISVV Ltyq25**	ISVV	France, Sauternes	Europe	grapes, high sugar must	grape/wine	Europe oak/France grapes	na
L. thermotolerans	**ISVV Ltyq3**	ISVV	France, Sauternes	Europe	grapes, high sugar must	grape/wine	Europe oak/France grapes	na

L. thermotolerans	**ISVV Ltyq36**	ISVV	France, Sauternes	Europe	grapes, high sugar must	grape/wine	Europe oak/France grapes	na
L. thermotolerans	**JCB1**	ISVV	France, Sauternes	Europe	grapes, high sugar must	grape/wine	Domestic 1	na
L. thermotolerans	**KEH.34.B.3**	na	USA, Missouri, Ste. Genevieve	North America	grapes, fermentation	grape/wine	Canada trees	Freel et al. 2014 [b]
L. thermotolerans	**LEVULIA® ALCOMENO**	AEB	France, Burgundy	Europe	grapes, fermentation	grape/wine	Europe oak/France grapes	na
L. thermotolerans	**LL12_031**	LL	Canada	North America	*Quercus sp.*tree bark	plant	Canada trees	na
L. thermotolerans	**LL12_036**	LL	Canada	North America	*Acer sp.* bark	plant	Canada trees	na
L. thermotolerans	**LL12_040**	LL	Canada	North America	*Acer sp.* bark	plant	Mix Europe/N. America	na
L. thermotolerans	**LL12_041**	LL	Canada	North America	*Quercus sp.*bark	plant	Mix Europe/N. America	na
L. thermotolerans	**LL12_056**	LL	Canada	North America	planted *Quercus sp.*bark	plant	Canada trees	na
L. thermotolerans	**LL13-038**	LL	USA, Massachusetts, Woburn	North America	*Quercus sp.*bark	plant	Mix Europe/N. America	na
L. thermotolerans	**LL13-171**	LL	Canada, New-Brunswick, Oak Point Provincial Park	North America	*Quercus sp.*bark	plant	Canada trees	na
L. thermotolerans	**LL13-175**	LL	Canada, New-Brunswick, Oak Point Provincial Park	North America	*Quercus sp.*bark	plant	Canada trees	na
L. thermotolerans	LL13-178	LL	Canada, New-Brunswick, Oak Point Provincial Park	North America	*Quercus sp.*bark	plant	Canada trees	na
L. thermotolerans	LL13-179	LL	Canada, New-Brunswick, Oak Point Provincial Park	North America	*Quercus sp.*bark	plant	Canada trees	na
L. thermotolerans	**LL13-189**	LL	Canada, New-Brunswick, Oak Point Provincial Park	North America	*Quercus sp.*bark	plant	Canada trees	na
L. thermotolerans	LL13-192	LL	Canada, New-Brunswick, Oak Point Provincial Park	North America	*Quercus sp.*bark	plant	Canada trees	na
L. thermotolerans	**LL13-194**	LL	Canada, New-Brunswick, Oak Point Provincial Park	North America	*Quercus sp.*bark	plant	Canada trees	na
L. thermotolerans	LL13-198	LL	Canada, New-Brunswick, Oak Point Provincial Park	North America	*Quercus sp.*bark	plant	Canada trees	na
L. thermotolerans	LL13-199	LL	Canada, New-Brunswick, Oak Point Provincial Park	North America	*Quercus sp.*bark	plant	Canada trees	na
L. thermotolerans	MB10D.1	NCYC	France, Montbarri	Europe	*Quercus sp.*	plant	Europe oak/France grapes	Robinson et al. 2016 [d]
L. thermotolerans	MB15D.1	NCYC	France, Montbarri	Europe	*Quercus sp.*	plant	Europe oak/France grapes	Robinson et al. 2016 [d]

L. thermotolerans	MELODY™	CHR Hansen	Mediterranean country''	na	na	na	Domestic 1	na
L. thermotolerans	**MUCL 31341**	MUCL	Italy	Europe	wine	grape/wine	Domestic 1	Freel et al. 2014 [b]
L. thermotolerans	**MUCL 31342**	MUCL	Italy	Europe	wine	grape/wine	Domestic 1	na
L. thermotolerans	MUCL 31343	MUCL	Italy	Europe	grapes, fermentation	grape/wine	Domestic 1	na
L. thermotolerans	MUCL 31349	MUCL	Italy	Europe	wine	grape/wine	Domestic 1	na
L. thermotolerans	**MUCL 47720**	MUCL	Italy	Europe	wine	grape/wine	Domestic 1	na
L. thermotolerans	**NCAIM Y.00775**	NCAIM	Hungary, Babat	Europe	*Carpinus betulu* exudate	plant	Mix Europe/N. America	na
L. thermotolerans	**NCAIM Y.00798**	NCAIM	Hungary, Csikóváralja	Europe	brown rotten *Quercus sp.*	plant	Mix Europe/N. America	na
L. thermotolerans	**NCAIM Y.00873**	NCAIM	Hungary, Budapest	Europe	rotten material of a cavity of *Betula pendula*	plant	Mix E. Europe	na
L. thermotolerans	**NCAIM Y.01703**	NCAIM	Hungary, Nagyeged	Europe	grapes	grape/wine	Mix Europe/N. America	na
L. thermotolerans	*NEM 1*	ITAP-DEMETER	Greece	Europe	grapes	grape/wine	Domestic 1	na
L. thermotolerans	*NEM 12*	ITAP-DEMETER	Greece	Europe	grapes	grape/wine	Mix Europe/N. America	na
L. thermotolerans	*NEM 3*	ITAP-DEMETER	Greece	Europe	grapes	grape/wine	Domestic 1	na
L. thermotolerans	*NEM 5*	ITAP-DEMETER	Greece	Europe	grapes	grape/wine	Domestic 1	na
L. thermotolerans	*NEM 6*	ITAP-DEMETER	Greece	Europe	grapes	grape/wine	Domestic 1	na
L. thermotolerans	*NEM 7*	ITAP-DEMETER	Greece	Europe	grapes	grape/wine	Domestic 1	na
L. thermotolerans	**NRLL Y-2193**	NRRL/ARS	USA, San Jacinto, California	North America	*Drosophila pseudoobscura*	insect	Americas	na
L. thermotolerans	**NRLL Y-2196**	NRRL/ARS	USA, San Jacinto, California	North America	*Drosophila pseudoobscura*	insect	Hawaii/California	na
L. thermotolerans	**NRLL Y-2197**	NRRL/ARS	USA, San Jacinto, California	North America	*Drosophila pseudoobscura*	insect	Americas	na
L. thermotolerans	**NRLL Y-27329**	NRRL/ARS	USA, West Virginia	North America	grapes	grape/wine	Domestic 2	na
L. thermotolerans	**NRLL Y-27911**	NRRL/ARS	USA, Louisiana	North America	gut of a fishfly	insect	Americas	Freel et al. 2014 [b]
L. thermotolerans	**NRLL Y-27937**	NRRL/ARS	USA, Louisiana	North America	surface of fishfly	insect	Canada trees	Freel et al. 2014 [b]
L. thermotolerans	NRLL YB-3379	NRRL/ARS	USA, Marion, Illinois	North America	rotten log	plant	Domestic 2	na
L. thermotolerans	**NZ156**	CRPR	New Zealand	Australia/Oceania	grapes cv. Chardonnay	grape/wine	Domestic 1	na
L. thermotolerans	**OCK6C.1**	NCYC	UK, Ocknell, New Forest	Europe	*Quercus sp.*	plant	Europe oak/France grapes	Robinson et al. 2016 [d]
L. thermotolerans	**OSU A**	OSU	USA, Oregon	North America	grapes	grape/wine	Mix Europe/N. America	na
L. thermotolerans	**PLU5B.1**	NCYC	UK, Plumpton vineyard	Europe	*Quercus sp.*	plant	Europe oak/France grapes	Robinson et al. 2016 [d]
L. thermotolerans	**PYR14B.1**	NCYC	Greece, Pyradikia	Europe	*Quercus sp.*	plant	Domestic 1	Robinson et al. 2016 [d]
L. thermotolerans	**T 13/17 F**	University of the Republic	Uruguay	South/Central America	grapes cv. Tannat	grape/wine	Domestic 2	na

L. thermotolerans	**TAX9D.1**	NCYC	Greece, Taxiarchis	Europe	*Quercus sp.*	plant	Mix Europe/N. America	Robinson et al. 2016 [d]
L. thermotolerans	**UNIFG 26**	UNIFG	Italy	Europe	wine	grape/wine	Domestic 1	na
L. thermotolerans	**UNIFG 28**	UNIFG	Italy	Europe	wine	grape/wine	Domestic 2	na
L. thermotolerans	**UNIFG 16**	UNIFG	Italy	Europe	wine	grape/wine	Domestic 2	na
L. thermotolerans	**UNIFG 17**	UNIFG	Italy	Europe	wine	grape/wine	Domestic 2	na
L. thermotolerans	**UNIFG 18**	UNIFG	Italy	Europe	wine	grape/wine	Domestic 2	na
L. thermotolerans	**UNIFG 22**	UNIFG	Italy	Europe	wine	grape/wine	Domestic 2	na
L. thermotolerans	**UNIFG 32**	UNIFG	Italy	Europe	wine	grape/wine	Domestic 2	na
L. thermotolerans	UNIFG 33	UNIFG	Italy	Europe	wine	grape/wine	Domestic 2	na
L. thermotolerans	**UWOPS 79-110**	UWOPS	Canada, Ontario	North America	black knot, *Prunus virginiana*	plant	Canada trees	Freel et al. 2014 [b]
L. thermotolerans	**UWOPS 79-116**	UWOPS	Canada, Pinery	North America	black knot, *Prunus virginiana*	plant	Canada trees	Freel et al. 2014 [b]
L. thermotolerans	**UWOPS 79-117**	UWOPS	Canada, Pinery	North America	black knot, *Prunus virginiana*	plant	Mix Europe/N. America	Freel et al. 2014 [b]
L. thermotolerans	UWOPS 79-162	UWOPS	Canada, Ontario, Melbourne	North America	black knot, *Quercus rubra*	plant	Mix Europe/N. America	Freel et al. 2014 [b]
L. thermotolerans	**UWOPS 79-164**	UWOPS	Canada, Ontario, Melbourne	North America	black knot, *Prunus virginiana*	plant	Canada trees	Freel et al. 2014 [b]
L. thermotolerans	**UWOPS 79-195**	UWOPS	Canada, Ontario, Melbourne	North America	black knot, *Prunus virginiana*	plant	Canada trees	Freel et al. 2014 [b]
L. thermotolerans	UWOPS 79-248	UWOPS	Canada, Ontario	North America	frass, Birch	plant	Mix Europe/N. America	Freel et al. 2014 [b]
L. thermotolerans	**UWOPS 79-255**	UWOPS	Canada, Ontario	North America	black knot, *Prunus pumila*	plant	Canada trees	Freel et al. 2014 [b]
L. thermotolerans	UWOPS 80-19	UWOPS	Canada, Ontario	North America	black knot, *Prunus virginiana*	plant	Canada trees	Freel et al. 2014 [b]
L. thermotolerans	**UWOPS 83-1097.1**	UWOPS	Cayman Islands, Cayman Brac	South/Central America	*Gitona americana*, *Opuntia stricta*	plant	Americas	Freel et al. 2014 [b], Naumova et al. 2007 [c]
L. thermotolerans	**UWOPS 83-1101.1**	UWOPS	Cayman Islands, Cayman Brac	South/Central America	*Gitona americana*, *Opuntia stricta*	insect	Americas	Freel et al. 2014 [b]
L. thermotolerans	**UWOPS 85-312.1**	UWOPS	USA, Arizona, Tuscon	North America	*Drosophila carbonaria, Prosopis juliflora*	insect	Americas	Freel et al. 2014 [b]
L. thermotolerans	**UWOPS 85-51.1**	UWOPS	USA, Florida, Big Pine Key	North America	*Opuntia cubensis*	plant	Americas	Freel et al. 2014 [b]
L. thermotolerans	**UWOPS 90-10.1**	UWOPS	Bahamas, Exumas Cays, Shroud Cay	South/Central America	Columnar cactus	plant	Americas	Freel et al. 2014 [b]
L. thermotolerans	UWOPS 90-1020.1	UWOPS	Bahamas, Exumas Cays, Shroud Cay	South/Central America	coco plum	plant	Americas	Freel et al. 2014 [b]
L. thermotolerans	UWOPS 91-902.1	UWOPS	Hawaii, Saddle Rd Park	Australia/Oceania	flux (white), *Myoporum*	plant	Hawaii/California	Freel et al. 2014 [b]
L. thermotolerans	**UWOPS 91-910.1**	UWOPS	Hawaii, Saddle Rd Park	Australia/Oceania	flux (pink), *Myoporum*	plant	Hawaii/California	Freel et al. 2014 [b]
L. thermotolerans	**UWOPS 91-912.1**	UWOPS	Hawaii, Saddle Rd Park	Australia/Oceania	flux (white), *Myoporum*	plant	Hawaii/California	Freel et al. 2014 [b]

L. thermotolerans	**UWOPS 94-426.2**	UWOPS	Mexico, Jalisco	South/Central America	distillery, agave must	agriculture/food	Domestic 1	Freel et al. 2014 [b]
L. thermotolerans	YJS4269	na	Ukraine	Europe	*Drosophila sp.*	insect	Americas	na
L. thermotolerans	*Y1017*	IWBT	South Africa	Africa	grapes cv. Chardonnay	grape/wine	Domestic 2	na
L. thermotolerans	*Y1038*	IWBT	South Africa	Africa	grapes cv. Chardonnay	grape/wine	Domestic 2	na
L. thermotolerans	*Y1109*	IWBT	South Africa	Africa	grapes cv. Sauvignon blanc	grape/wine	Domestic 2	na
L. thermotolerans	*Y1202*	IWBT	South Africa	Africa	grapes cv. Sauvignon blanc	grape/wine	Domestic 2	na
L. thermotolerans	*Y1206*	IWBT	South Africa	Africa	grapes cv. Sauvignon blanc	grape/wine	Domestic 2	na
L. thermotolerans	*Y1295*	IWBT	South Africa	Africa	grapes, fermentation (Sauvignon blanc – Chardonnay blend)	grape/wine	Domestic 2	na
L. thermotolerans	*Y905*	IWBT	South Africa	Africa	grapes cv. Chenin blanc	grape/wine	Domestic 2	na
L. thermotolerans	**Yal. 87-1**	na	Russia, Crimea	Russia/Asia	*Quercus sp.* exudate	plant	Europe oak/France grapes	Naumova et al. 2007 [c]
L. thermotolerans	**Yal. 87-2**	na	Finland	Europe	*Quercus sp.* exudate	plant	Other	Naumova et al. 2007 [c]
L. thermotolerans	Yal. 87-5	na	Russia, Crimea	Russia/Asia	*Quercus sp.* exudate	plant	Mix E. Europe	Naumova et al. 2007 [c]
L. thermotolerans	**ZIM 2492**	ZIM	Serbia	Europe	rasberries	agriculture/food	Domestic 1	na
L. thermotolerans	**ZIM 2505**	ZIM	Serbia	Europe	rasberries	agriculture/food	Mix Europe/N. America	na
L. cidri	**CBS 4575T**	CBS-KNAW	na	na	na	na	na	na
L. dasiensis	**CBS 10888T**	CBS-KNAW	na	na	na	na	na	Lee et al. 2009 [e]
L. fantastica	**CBS 6924T**	CBS-KNAW	na	na	na	na	na	na
L. fermentati	**CBS 707T**	CBS-KNAW	na	na	na	na	na	na
L. kluyverii	**CBS 3082T**	CBS-KNAW	na	na	na	na	na	na
L. lanzarotensis	**CBS 12615T**	CBS-KNAW	na	na	na	na	na	Gonzalez et al. 2013 [f]
L. meyersii	**CBS 8951T**	CBS-KNAW	na	na	na	na	na	Fell et al. 2004 [g]
L. mirantina	**CBS 11717T**	CBS-KNAW	na	na	na	na	na	Pereira et al. 2011 [h]
L. nothofagi	**CBS 11611T**	CBS-KNAW	na	na	na	na	na	Mestre et al. 2010 [i]
L. quebecensis	**CBS 14138T**	CBS-KNAW	na	na	na	na	na	Freel et al. 2016 [j]
L. waltii	**CBS 6430T**	CBS-KNAW	na	na	na	na	na	na

Phaff YCC – Phaff Yeast Culture Collection, University of Davis, California, USA; **AWMCC** - AWRI Wine Microorganism Culture Collection, Australia; **CBS-KNAW** - Centraalbureau voor Schimmelcultures – Koninklijke Nederlandse Akademie van Wetenschappen , Netherlands; **CRBOeno** - Centre de Ressources Biologiques OEnologie, France; **DBVPG** - The Industrial Yeasts Collection DBVPG, Italy; **NCYC** - National Collection of Yeast Cultures, UK;, Italy; **BOKU** - Universität für Bodenkultur Wien, Austria; **ISVV** - Institut des Sciences de la Vigne et du Vin, France; **LL** – Landry Lab, Canada; **MUCL** - Mycothèque de l'Université

catholique de Louvain, Belgium; **NCAIM** - National Collection of Agricultural and Industrial Microorganisms, Hungary; **ITAP-DEMETER** - Institute of Technology of Agricultural Products, Hellenic Agricultural Organisation, Greece; **NRRL/ARS** - NRRL Agriculture Research Service culture collection, USA; **CRPR** - Centre de Recherche Pernod-Ricard, France; **OSU** – Oregon State University, USA; **UNIFG -** University of Foggia; **UWOPS** - Culture collection of the University of Western Ontario; **IWBT** – Institute for Wine Biotechnology, University of Stellenbosch, South Africa; **ZIM** - Zbirka industrijskih mikroorganizmov, Slovenia. [a] Sipiczki M. Overwintering of vineyard yeasts: Survival of interacting yeast communities in grapes mummified on vines. Frontiers in microbiology. 2016 Feb doi: 10.3389/fmicb.2016.00212; [c] Freel KC, Friedrich A, Hou J, Schacherer J. Population genomic analysis reveals highly conserved mitochondrial genomes in the yeast species *Lachancea thermotolerans*. Genome biology and evolution. 2014 Oct; 6(10):2586-94.; [c] Naumova ES, Serpova EV, Naumov GI. Molecular systematics of *Lachancea* yeasts. Biochemistry (Moscow). 2007 Dec; 72(12):1356-62.; [d] Robinson HA, Pinharanda A, Bensasson D. Summer temperature can predict the distribution of wild yeast populations. Ecology and evolution. 2016 Feb; 6(4):1236-50.; [e] Lee CF, Yao CH, Liu YR, Hsieh CW, Young SS. *Lachancea dasiensis* sp. nov., an ascosporogenous yeast isolated from soil and leaves in Taiwan. International journal of systematic and evolutionary microbiology. 2009 Jul; 59(7):1818-22.; [f] González SS, Alcoba-Flórez J, Laich F. *Lachancea lanzarotensis* sp. nov., an ascomycetous yeast isolated from grapes and wine fermentation in Lanzarote, Canary Islands. International journal of systematic and evolutionary microbiology. 2013 Jan; 63(1):358-63., [g] Fell JW, Statzell-Tallman A, Kurtzman CP. *Lachancea meyersii* sp. nov., an ascosporogenous yeast from mangrove regions in the Bahama Islands. Studies in Mycology 2004 Dec; 50:359-63.; [h] Pereira LF, Costa Jr CR, Brasileiro BT, de Morais Jr MA. *Lachancea mirantina* sp. nov., an ascomycetous yeast isolated from the cachaca fermentation process. International journal of systematic and evolutionary microbiology. 2011 Apr; 61(4):989-92.; [i] Mestre MC, Ulloa JR, Rosa CA, Lachance MA, Fontenla S. *Lachancea nothofagi* sp. nov., a yeast associated with *Nothofagus* species in Patagonia, Argentina. International journal of systematic and evolutionary microbiology. 2010 Sep; 60(9):2247-50.; [j] Freel KC, Charron G, Leducq JB, Landry CR, Schacherer J. *Lachancea quebecensis* sp. nov., a yeast species consistently isolated from tree bark in the Canadian province of Québec. International journal of systematic and evolutionary microbiology. 2015 Oct 1;65(10):3392-9.
