# Supplementary figures and images for: The evolution of Lachancea thermotolerans is driven by geographical determination, anthropisation and flux between different ecosystems
Source: PLoS One. 2017 Sep 14;12(9):e0184652. doi: 10.1371/journal.pone.0184652 (PMC5599012; doi:10.1371/journal.pone.0184652)

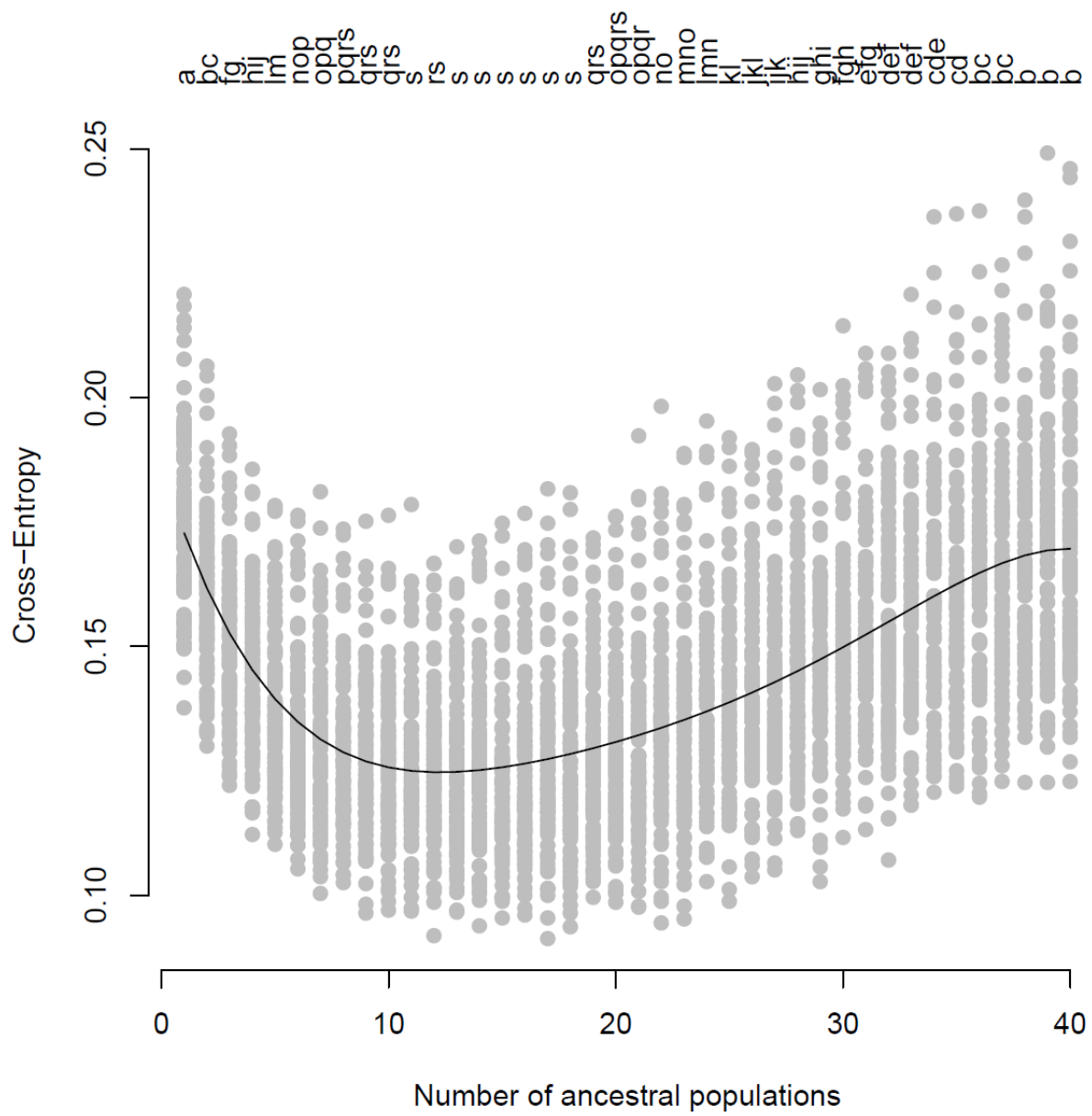

Supplement: S1 Fig — (PDF) [file pone.0184652.s003.pdf]
