# Supplementary material for: The evolution of Lachancea thermotolerans is driven by geographical determination, anthropisation and flux between different ecosystems
Source: PLoS One. 2017 Sep 14;12(9):e0184652. doi: 10.1371/journal.pone.0184652 (PMC5599012; doi:10.1371/journal.pone.0184652)
Supplement: S2 Table — Numbers are coded as following: 0—no amplification, 1—faint band, 2—medium intensity band, 3—full intensity band as determined using a microchip electrophoresis system. (PDF) [file pone.0184652.s002.pdf]

Species	Microsatellite loci													
	LT2A	LT3A	LT3B	LT4A	LT5B	LT6B	LT7A	LT8A	LT8B	MA2	MD3	ME11	ME4	MH6
L. cidri	0	3	2	0	3	1	2	0	2	0	1	1	1	0
L. dasiensis	1	2	1	0	3	3	1	0	2	0	0	0	1	0
L. fantastica	0	3	2	0	3	3	2	0	2	1	0	1	0	0
L. fermentati	0	2	2	0	3	2	1	0	0	0	1	0	1	0
L. kluyverii	0	0	1	0	2	3	0	0	2	1	0	1	1	0
L. lanzarotensis	2	2	0	0	1	3	0	1	0	0	0	2	0	0
L. meyersii	0	3	2	0	3	3	1	0	0	0	0	1	1	0
L. mirantina	0	2	0	0	3	3	2	0	3	0	0	0	1	1
L. nothofagi	0	2	0	0	3	3	1	0	2	0	3	1	1	0
L. quebecensis	0	2	1	0	3	3	0	0	1	3	0	1	0	1
L. waltii	0	2	1	0	1	3	1	0	1	0	0	2	1	0
L. thermotolerans	3	3	3	3	3	3	3	3	3	3	3	3	3	3
